# An N4-like *Caulobacter* phage requires host smooth lipopolysaccharide biosynthesis for infection

**DOI:** 10.1128/jb.00488-25

**Published:** 2026-02-25

**Authors:** Maeve McLaughlin, Katheren Barger, Charlotte Barron, Makena Fisher, Larissa Kohn, Priscilla Mac-Kittah, Aretha Fiebig, Sean Crosson

**Affiliations:** 1 Department of Biology, University of Michigan-Flint 14712, Flint, Michigan, USA; 2 Department of Microbiology, Genetics, and Immunology, Michigan State University 3078, East Lansing, Michigan, USA; University of California San Francisco, San Francisco, California, USA

**Keywords:** bacteriophage, lipopolysaccharide, *Caulobacter*, Tn-seq, polysaccharide, cell envelope, N4

## Abstract

*Caulobacter* species are Alphaproteobacteria that commonly inhabit plant-associated and aquatic microbial communities. Although *Caulobacter* is widespread and has long served as a model for the study of bacterial cell biology, our understanding of viruses that infect *Caulobacter* species remains limited. Here, we describe the discovery and characterization of Circe, a freshwater N4-like podophage belonging to the *Schitoviridae* family that infects *Caulobacter crescentus*. We isolated two variants, CirceC and CirceH, that differ by a single nucleotide, resulting in an F91I substitution in Gp063, an uncharacterized protein found in diverse bacteriophages and bacteria. While both Circe variants adsorb to *C. crescentus* with similar efficiency, they produce morphologically distinct plaques and display different infection dynamics. Through forward genetic selection and genome-wide transposon fitness profiling, we identified *C. crescentus* genes involved in cell envelope assembly, membrane sphingolipid biosynthesis, and envelope polysaccharide biosynthesis that influence susceptibility to Circe infection. Loss-of-function mutations in a predicted nucleoside diphosphate sugar epimerase and multiple genes required for smooth lipopolysaccharide (S-LPS) biosynthesis and export conferred strong resistance to infection. Furthermore, an in-frame deletion mutant defective in S-LPS production exhibited a strong defect in Circe adsorption. These results support a model in which S-LPS functions as a receptor for phage Circe. Our study expands the known repertoire of *Caulobacter* phages and adds to a growing understanding of the role of envelope polysaccharides in bacterial infection by N4-family phages.

**IMPORTANCE:**

Viruses that infect bacteria and archaea, known as phages, shape microbial communities through their effects on host survival and gene flow. Yet for many microbes, the phages that infect them and the host structures required for infection remain poorly characterized. *Caulobacter* species are ecologically important Alphaproteobacteria that can produce protein surface structures, such as pili, a flagellum, and an S-layer, which are established phage receptors in this genus. We discovered *Caulobacter* phage Circe and provided evidence that it relies on smooth lipopolysaccharide and other envelope carbohydrates to infect *Caulobacter crescentus*. This study broadens the understanding of phage–host interactions in *Caulobacter* and establishes Circe as a useful system for dissecting how phages engage with bacterial cells.

## INTRODUCTION

*Caulobacter* is a widely distributed genus of Alphaproteobacteria found in diverse environments, including aquatic habitats, soils, and plant-associated niches (1, 2). Long studied as a model system for bacterial cell biology (3), *Caulobacter* spp. are now also recognized as important members of plant-associated microbiomes (4) with select strains shown to promote plant growth (5, 6). The diverse ecological roles of *Caulobacter*, together with its genetic tractability, make it a powerful system for studying how environmental interactions impact bacterial biology.

Understanding how *Caulobacter* interacts with its environment requires consideration of the biotic forces that shape its physiology, ecology, and evolution. Among the most significant of these are bacteriophages, which are viruses that infect bacteria. With an estimated global abundance of approximately 10³¹ particles, bacteriophages (or phages) are the most numerous biological entities on Earth (7). Phages play critical roles in microbial communities by regulating host population sizes, driving nutrient turnover, and mediating genetic exchange in communities (8–11). Infection of a host cell by a phage typically begins with highly specific binding to a surface receptor molecule, such as a protein or polysaccharide. These receptor-mediated interactions exert intense selective pressure on bacterial populations and frequently lead to the emergence of phage-resistant mutants with altered cell surface properties (12–14). Several *Caulobacter*-infecting phages are known. Among them, the virulent siphophage φCbK was one of the first reported (15) and has proven a valuable tool for studying polar morphogenesis, as infection requires the polar flagellum and pili (16); it has more recently emerged as a model for the study of *Caulobacter*-phage regulatory cross-talk during infection (17). The T4-like phage, φCr30, uses the paracrystalline surface-layer protein as a receptor (18) and remains a useful tool for transduction of *Caulobacter* (19). Other double-stranded DNA and RNA *Caulobacter* phages have also been described (20–22), but the broader diversity and ecological roles of *Caulobacter* phages remain poorly defined.

The N4-like group of phages represents a distinct viral lineage that was first isolated on *Escherichia coli* in 1966 (23). Phage N4 is a podovirus characterized by an icosahedral capsid, a short non-contractile tail, and a linear double-stranded DNA genome of approximately 70 kb (24, 25). A defining feature of this group is a large virion-associated RNA polymerase (vRNAP), which is co-ejected with the genome to initiate early transcription in the host cell (26–29). N4 coliphage is also notable for its delayed lysis strategy, which enables exceptionally high burst sizes, often exceeding 3,000 plaque-forming units (PFU) per infected cell (30).

Here, we report the discovery and characterization of Circe, an N4-like phage of the *Schitoviridae* family that infects *Caulobacter crescentus*. To our knowledge, Circe is the first N4-like phage described to infect a *Caulobacter* species. We isolated two phage variants, CirceC and CirceH, that differ by only a single nucleotide yet have distinct plaque phenotypes and infection kinetics in a broth infection assay. Through a combination of genetic selections and screening approaches, we demonstrate that successful infection by Circe requires several host genes that function in cell envelope carbohydrate metabolism. Specifically, we identify a WcaG-like NAD-dependent sugar epimerase and multiple genes required for lipopolysaccharide (LPS) O-polysaccharide biosynthesis as key host factors for Circe infection. These results expand our understanding of N4-like phage biology, reveal new molecular determinants of phage susceptibility in *C. crescentus*, and establish Circe as a tractable model for studying phage–host interactions in an ecologically important genus.

## RESULTS

### Isolation and structural characterization of *Caulobacter* phages

While measuring *C. crescentus* CB15 growth in filtered water collected from an inland freshwater lake in Haslett, Michigan (USA), we unexpectedly observed zones of clearing on spot plates used for colony-forming unit enumeration. These clearing zones were consistent with lytic bacteriophage activity and were sampled for further analysis. Subsequent experiments confirmed that the clearing was caused by a phage capable of infecting and lysing *C. crescentus* CB15 on solid medium.

**Fig 1:**
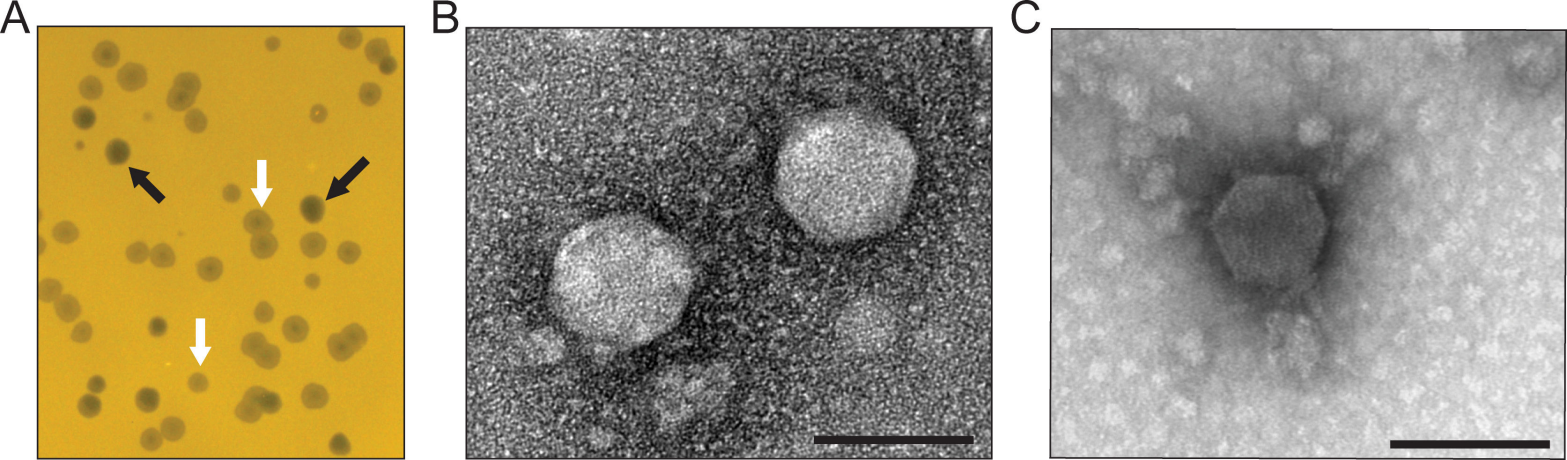
Isolation and structural characterization of *Caulobacter*-infecting phages from Lake Lansing (Michigan, USA). (**A**) Representative plaque morphologies formed on *C. crescentus* CB15 lawns grown on peptone yeast extract (PYE) agar. Two reproducible plaque types were observed: clear plaques (black arrows) and halo plaques with turbid outer rings (white arrows), consistent with distinct phage variants. (**B–C**) Transmission electron micrographs of negatively stained phage particles purified from clear plaques (**B**) and halo plaques (**C**), both displaying podovirus morphology characterized by icosahedral capsids and short tails. Scale bars: 100 nm.

Two distinct plaque morphologies were observed: (i) clear plaques and (ii) halo plaques characterized by a clear center and turbid outer edge (Fig. 1A). Phage isolated from each plaque type consistently reproduced the original plaque morphology, suggesting that the clear and halo plaques were generated by distinct phages. We purified and imaged phage particles from each plaque type using transmission electron microscopy (TEM). The phage from the clear plaques and the phage from the halo plaques both displayed capsids with mean diameters of 79.8 ± 5.4 nm (*n* = 18) and 69.5 ± 3.5 nm (*n* = 17), respectively (Fig. 1B and C). Neither phage exhibited a prominent tail structure.

### Genomic features of clear- and halo plaque-forming variants of *Caulobacter* phage Circe

We isolated and purified phages from clear and halo plaques and sequenced their genomes. Both phage genomes comprised a 73,793 bp linear double-stranded DNA genome (GenBank accession PX630703.1) with 341 bp direct terminal repeats and a G+C content of 53%, which is notably lower than that of the *C. crescentus* host genome (67.2%; GenBank accession CP001340). There was only a single base pair difference between the clear plaque- and halo plaque-forming phage: an A-to-T transversion at position 38,705 in the isolate that forms halo plaques. Genome annotation (see Materials and Methods) predicted 100 open reading frames (ORFs) and 2 tRNA genes (Table S1). Accordingly, we designate the halo plaque-forming isolate *Caulobacter* phage CirceH and the clear plaque-forming isolate *Caulobacter* phage CirceC.

**Fig 2:**
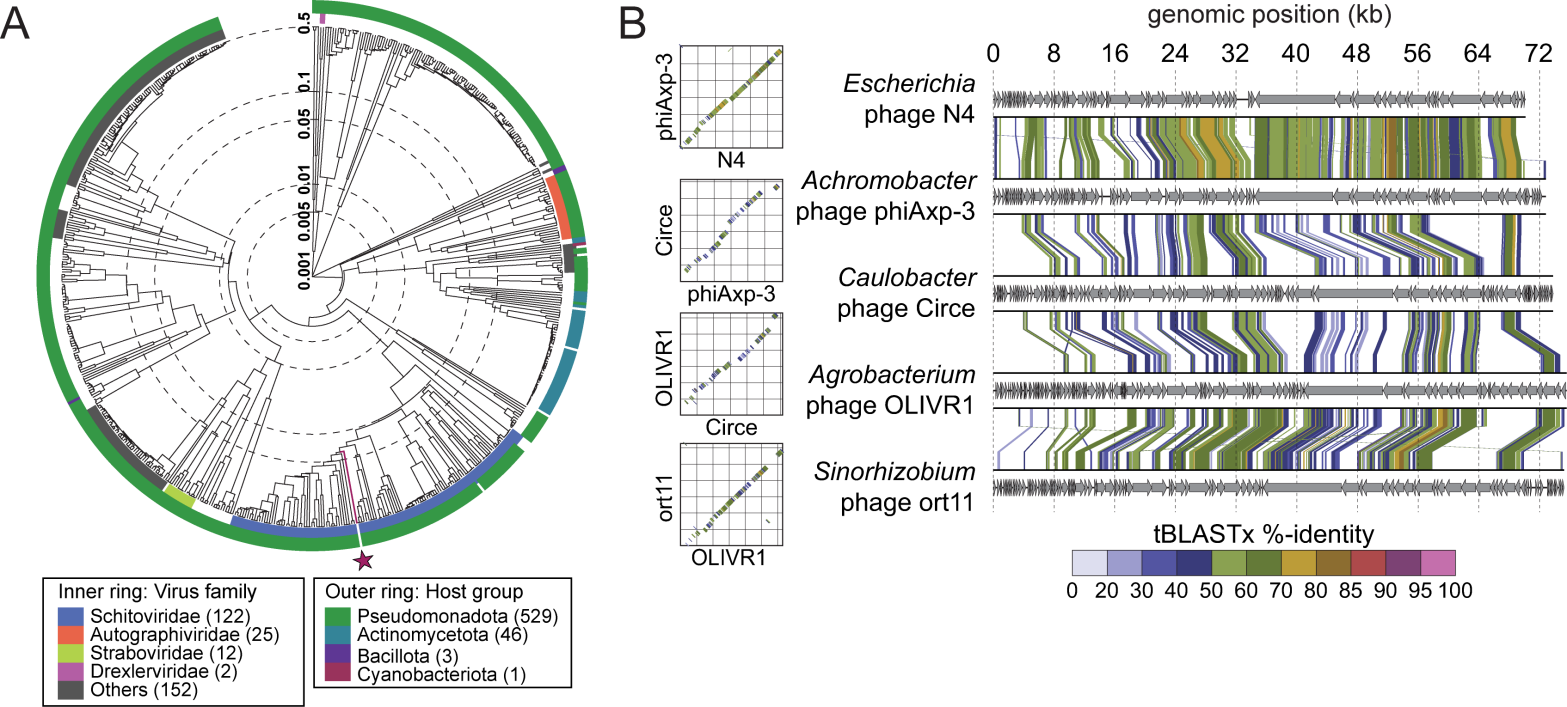
Circe is a member of the N4-like Schitoviridae family. (**A**) Viral proteomic tree generated using ViPTree (http://www.genome.jp/viptree) showing the relationship between Circe and 590 related phage genomes. The inner ring indicates viral family-level classification, and the outer ring indicates host taxonomic group. Circe is marked with a red line and a red star. (**B**) Genome comparisons between Circe and representative N4-like phages. Linear genome alignments (right) and dot plots (left) are shown, with arrows indicating predicted genes and connecting lines denoting regions of nucleotide identity identified by tBLASTx. Color corresponds to percent identity (as labeled) and highlights synteny homology across the N4-like lineage.

Phylogenetic analysis (31) placed Circe within the *Schitoviridae* family, clustering it with N4-like phages (Fig. 2A; Fig. S1). The N4 phages are defined by the presence of seven hallmark genes (25): a DNA polymerase, a large vRNAP, a portal protein, a major capsid protein, a tail protein, and both the large and small terminase subunits; Circe contains all seven hallmark genes and exhibits synteny homology with other N4 genomes (Fig. 2B). Analysis of genes associated with host cell lysis revealed four ORFs with predicted roles in this process. Among these was *gp077*, predicted to encode an Rz-like inner membrane spanin (i-spanin). An outer membrane spanin (o-spanin) was not initially evident based on automated gene calling approaches, but a *tblastn* search using the o-spanin from *Escherichia* phage N4 identified a candidate gene in the +1 reading frame relative to *gp077*. This gene, designated *gp078*, shared 52% overall identity with the periplasmic domain of the N4 o-spanin and possessed a predicted outer membrane lipoprotein signal sequence, supporting its identity as a Circe o-spanin. Downstream of the spanin system, *gp079* was annotated as a glycoside hydrolase family endolysin (IPR002196), likely involved in peptidoglycan degradation. While no holin or antiholin genes were definitively identified, three downstream ORFs (*gp080–gp082*) encoded predicted transmembrane domains, raising the possibility that they function in membrane disruption during lysis. As noted above, the only genomic difference between CirceH and CirceC is a single A-to-T transversion at position 38,705 in CirceH, resulting in a phenylalanine-to-isoleucine (F91I) substitution in *gp063*, a gene of unknown function. The mechanism by which this mutation may impact plaque morphology is discussed in a later section.

### Host genetic background and phage genetic variation impact infection phenotypes

**Fig 3:**
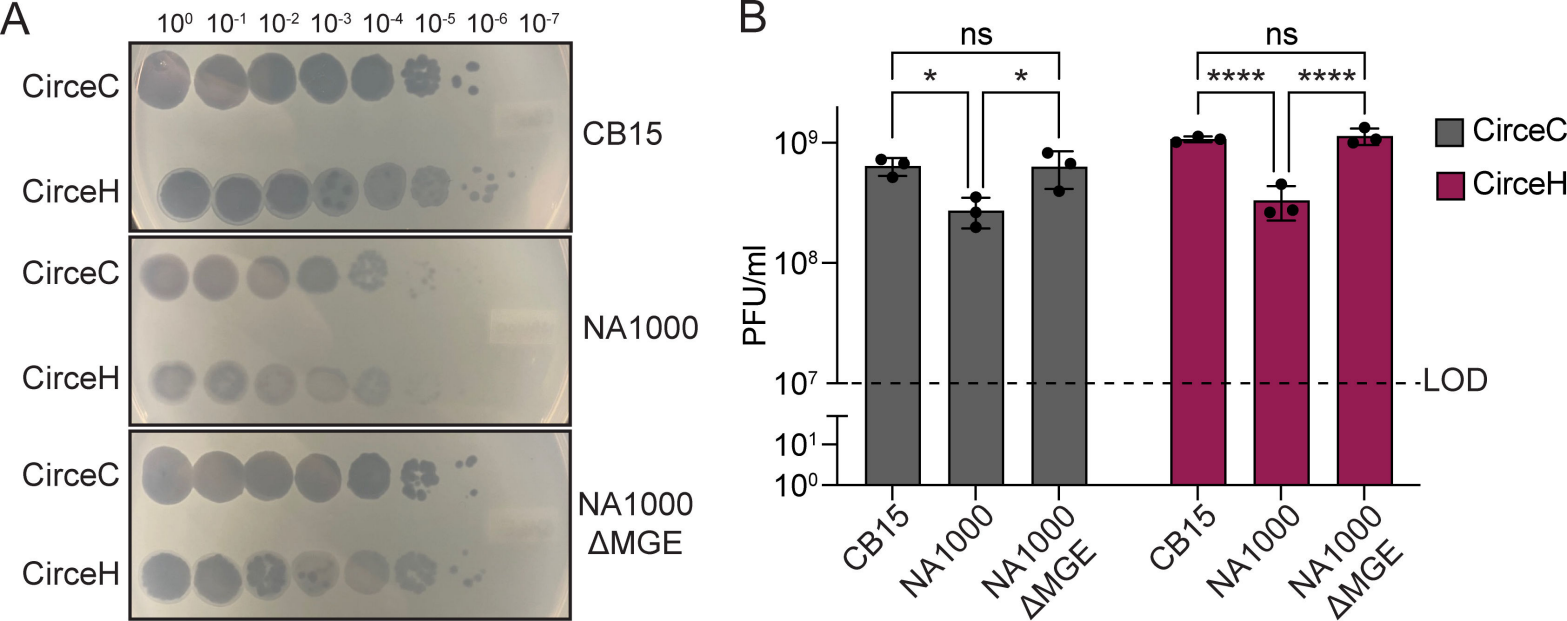
An MGE in *C. crescentus* NA1000 reduces susceptibility to phage Circe. (**A**) Ten-fold serial dilutions of CirceC (clear plaques) and CirceH (halo plaques) were spotted onto PYE top agar lawns of *C. crescentus* CB15 (top), NA1000 (middle), and an NA1000 derivative lacking a 26-kb mobile genetic element (NA1000 ΔMGE; bottom). Images are representative of three biological replicates. (**B**) EOP for CirceC and CirceH on strains in panel A. Bars show the mean ± SD from three biological replicates. The dashed line indicates the limit of detection (LOD). Statistical significance was assessed by two-way ANOVA with Šídák’s multiple-comparisons test (ns, not significant; *, *P* < 0.05; ****, *P* < 0.0001).

To evaluate the infection dynamics of the two Circe variants in closely related *C. crescentus* strains with distinct cell envelope features, we measured the efficiency of plating (EOP) of CirceC (clear plaque-forming) and CirceH (halo plaque-forming) on strain NA1000. NA1000 is a laboratory strain that is nearly identical to CB15; the two strains descend from a common ancestral isolate (32) and differ by only eight single-nucleotide polymorphisms, two single-base insertions or deletions, and the presence of a 26-kb mobile genetic element (MGE) (32). Compared to the *C. crescentus* CB15 strain, both CirceC and CirceH formed fewer and more turbid plaques on NA1000 (Fig. 3), suggesting reduced infectivity. Previous work has shown that the MGE in strain NA1000 encodes a suite of enzymes involved in exopolysaccharide (EPS) biosynthesis, and that this EPS production enhances resistance to the S-layer-targeting phage φCr30 (32–34). To test whether the MGE also affects susceptibility to CirceC and CirceH infection, we examined EOP on an NA1000 derivative lacking the MGE (ΔMGE) (32). On the NA1000 ΔMGE strain, both phages formed plaques comparable in number and clarity to those observed on CB15; thus, loss of the MGE enhances phage susceptibility. We conclude that MGE-encoded EPS biosynthetic processes contribute to resistance to Circe infection.

**Fig 4:**
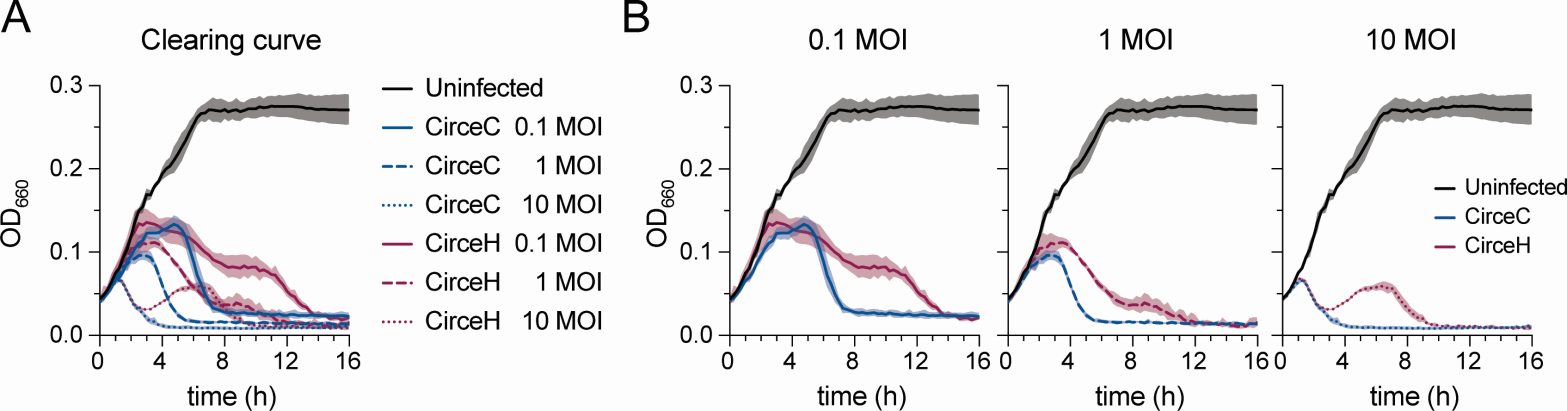
CirceC and CirceH exhibit distinct clearing kinetics during infection of *C. crescentus*. (**A**) *C. crescentus* CB15 broth cultures infected with either CirceC or CirceH at MOIs of 0.1, 1, or 10 in complex PYE medium. Optical density at 660 nm (OD₆₆₀) was measured every 15 min for 16 h to track culture clearing dynamics. Uninfected cultures served as controls. (**B**) Data from panel A are reorganized by MOI (bottom row: 0.1, 1, 10) to highlight kinetic differences. Lines represent the mean, and shaded areas represent the standard deviation from three biological replicates.

We next compared the ability of CirceC and CirceH to infect and lyse *C. crescentus* in liquid culture. We infected exponentially growing CB15 cells at multiplicities of infection (MOIs) of 0.1, 1, and 10 and monitored optical density at 660 nm over time (Fig. 4). Infections with CirceC produced a characteristic delay in growth suppression, followed by a rapid decline in culture density, consistent with lytic activity. The timing of clearing, evidenced by a decline in culture density, was MOI-dependent: clearing began approximately 5 h post-infection at MOI 0.1, 3 h at MOI 1, and within 1 h at MOI 10. In contrast, CirceH infections followed a more complex pattern. While culture clearing occurred at all MOIs, the decline in optical density was slower and biphasic. At MOI 10, absorbance initially decreased from 1 to 3 h, then partially rebounded from 3 to 6.5 h before declining again and fully clearing by 10 h (Fig. 4). Similar delayed or multiphasic trends were observed at lower MOIs. These results suggest that despite near-identical genomes, CirceH exhibits altered infection dynamics compared to CirceC, possibly reflecting differences in adsorption efficiency, replication kinetics, or interactions with host cell surface structures.

### CirceC and CirceH have equivalent bulk adsorption properties

**Fig 5:**
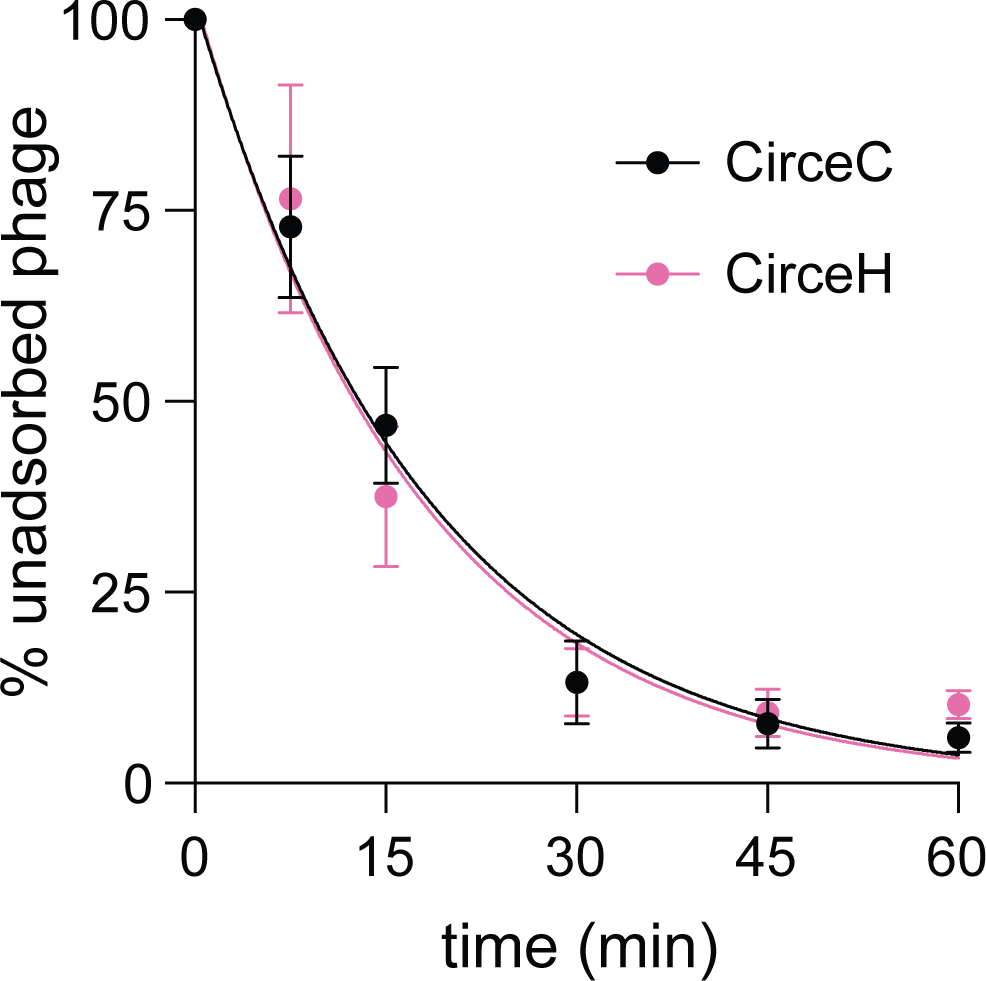
Adsorption of CirceC and CirceH to *C. crescentus* CB15. Exponential phase *C. crescentus* CB15 cultures infected with either CirceC (black circles) or CirceH (pink circles) at an MOI of 0.01 in PYE medium. At the indicated time points post-infection, the fraction of unadsorbed phage in the supernatant was quantified and expressed as a percentage relative to the initial phage titer at time zero. Data represent the mean ± standard deviation of three biological replicates. Curves represent fits to the model: *P*(*t*) = *P*₀ × exp(–*k* × *n* × *t*), where *P(t*) is the concentration of unadsorbed phage at time *t*, *P₀* is the initial phage concentration, *n* is the bacterial cell density, *t* is time (min), and *k* is the adsorption rate constant. The R² goodness of fit for each regression: CirceH = 0.94; CirceC = 0.97.

We postulated that the differences in infection dynamics between the two Circe variants were due to differences in their ability to adsorb to host cells. We therefore quantified adsorption of CirceC and CirceH to *C. crescentus* CB15 over a 60-min time course by measuring the fraction of unadsorbed phage remaining in culture supernatants. Both variants displayed similar adsorption properties (Fig. 5). For CirceC, approximately 87% of phage particles were adsorbed within 30 min of incubation, and adsorption reached near completion by 60 min, yielding an adsorption rate constant of k = 4.2 × 10⁻¹⁰ mL cell^−1^ min⁻¹. CirceH had a comparable profile, with 86% adsorption by 30 min and a calculated rate constant of k = 4.3 × 10⁻¹⁰ mL cell^−1^ min⁻¹. We conclude that the F91I substitution in Gp063 that distinguishes CirceH from CirceC does not measurably alter the efficiency or kinetics of phage adsorption to *C. crescentus* CB15.

### Loss-of-function mutations in a WcaG-like epimerase gene confer resistance to Circe

**Fig 6:**
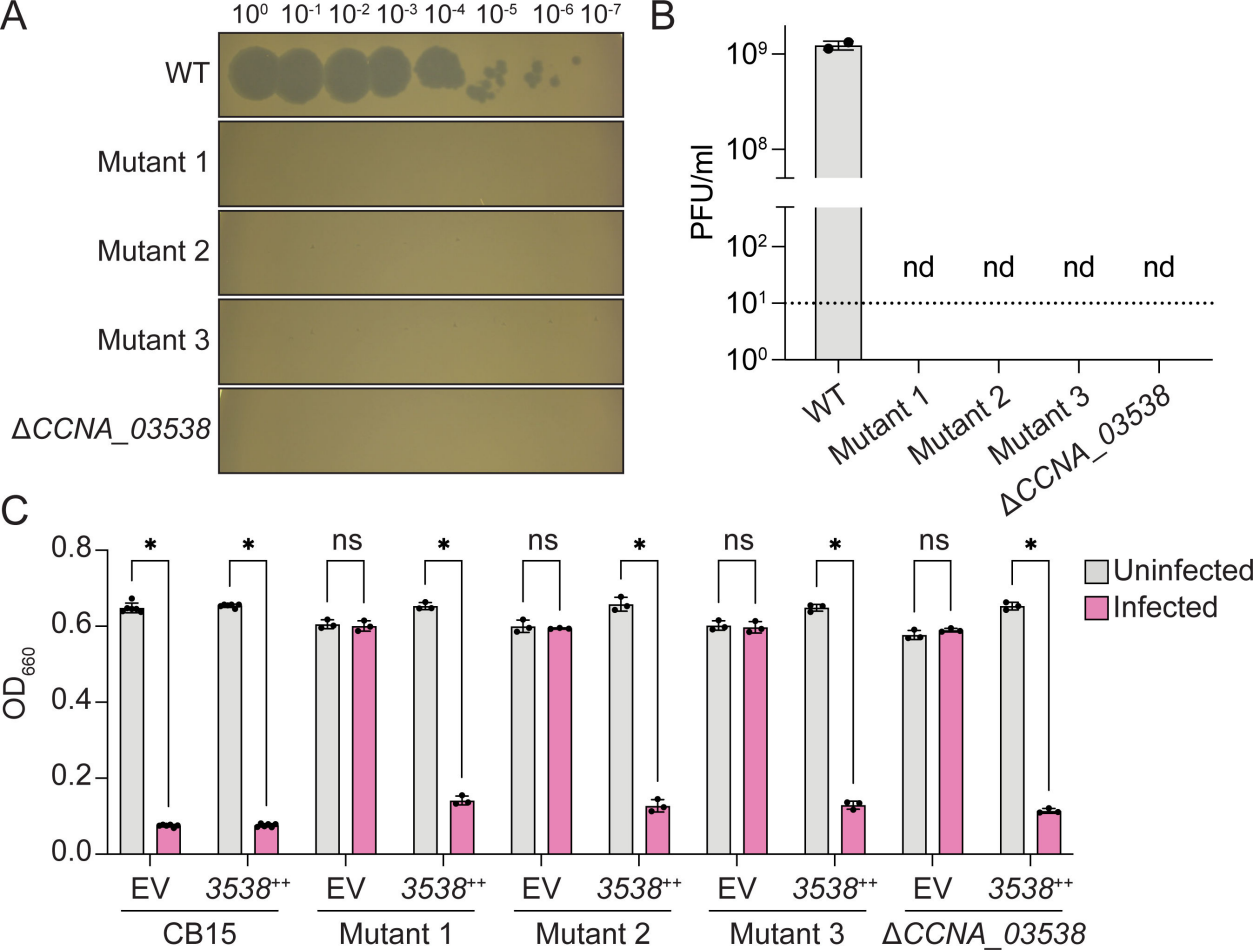
Mutation or deletion of a putative WcaG-family epimerase (*CCNA_03538*) confers resistance to Circe infection. (**A**) Plaque assay showing CirceC infection of wild-type *C. crescentus* CB15, three spontaneous Circe-resistant mutants (mutants 1–3), and an in-frame *CCNA_03538* deletion strain (Δ*CCNA_03538*). Ten-fold serial dilutions of CirceC were spotted onto PYE top agar lawns of the indicated strains. (**B**) EOP for CirceC on strains in panel** A**. Dashed line indicates the LOD, and nd indicates no plaques detected. (**C**) Growth of liquid PYE cultures infected with CirceC (MOI 0.1; pink) or mock-infected (gray), measured as OD₆₆₀ after 24 h. Strains carried either an empty vector (EV) or a plasmid expressing *CCNA_03538* from an inducible promoter (*3538*++). Bars represent the mean ± SD from two (**B**) or three (**C**) biological replicates. Statistical significance was assessed by multiple unpaired *t*-tests with Holm–Šídák correction for multiple comparisons (ns, not significant; *P < 0.05).

In the process of conducting plaque assays with CirceC, we observed that *C. crescentus* colonies occasionally emerged within lysis zones after 2 days of incubation. To test whether these colonies were resistant to CirceC, we isolated three independent clones and measured their susceptibility to infection. All three mutants exhibited complete resistance to CirceC: no plaques were observed at any phage concentration (Fig. 6A and B), and cultures inoculated with 0.1 MOI of CirceC grew comparably to uninfected controls in liquid culture (Fig. 6C). To identify the genetic basis for resistance, we sequenced the genomes of these three spontaneous mutants and compared them to the parental *C. crescentus* CB15 strain. Each mutant harbored a distinct mutation in a gene encoding a WcaG-like NAD-dependent epimerase (gene locus tag *CCNA_03538* or *CC_3425*) (Table S2). Hereafter, we refer to *C. crescentus* gene locus tags using the CCNA_ prefix, which corresponds to the curated *C. crescentus* genome annotation. The mutations in CCNA_03538 result in a premature stop (Y249*), a frameshift in the first third of the gene (∆1 bp at 298/870 bp), or a non-synonymous change (M260K).

To test whether *CCNA_03538* is necessary for CirceC infection, we generated an in-frame deletion strain (Δ*CCNA_03538*) and assessed its phage sensitivity. Like the spontaneous mutants, Δ*CCNA_03538* was completely resistant to CirceC infection in both plaque and liquid assays (Fig. 6A). Genetic complementation of either the in-frame deletion strain or the spontaneous mutants with *CCNA_03538* expressed from a cumate-inducible plasmid (35) restored phage susceptibility, confirming that loss of *CCNA_03538* function confers CirceC resistance (Fig. 6C).

### Genome-wide identification of Circe host factors by RB-TnSeq

**Fig 7:**
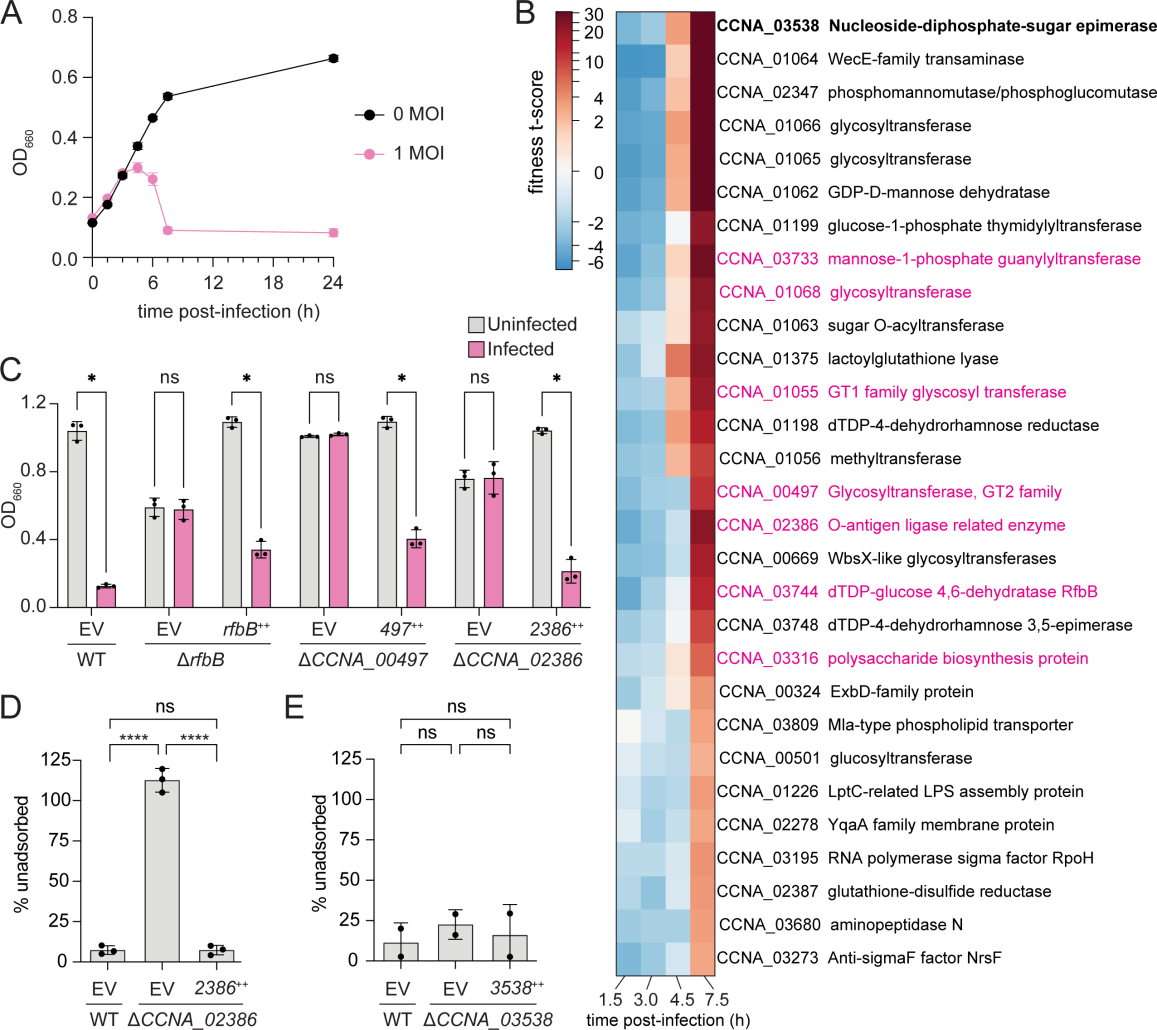
Disruption of smooth LPS and other cell envelope polysaccharide genes confers resistance to Circe infection. (**A**) Growth of a *C. crescentus* CB15 RB-TnSeq library in the presence or absence of CirceC. Exponential-phase cultures in PYE were infected at MOI 1 (pink) and monitored by OD₆₆₀ over 24 h. (**B**) Hierarchically clustered heatmap excerpt showing gene fitness *t*-scores at 1.5, 3.0, 4.5, and 7.5 h post-infection (cutoff: |*t|* ≥ 4 at ≥1 time point). The highlighted cluster (pink) contains genes associated with S-LPS biosynthesis. Positive scores indicate increased mutant fitness during infection; negative scores indicate decreased fitness. Additional genes meeting cutoff criteria are shown in Fig. S2, and complete fitness data are provided in Table S3. (**C**) Growth of *rfbB*, *CCNA_00497*, and *CCNA_02386* mutants with (pink) or without (gray) CirceC infection (MOI 0.1) in liquid PYE, measured as OD₆₆₀ after 24 h. (**D–E**) CirceC adsorption assays performed with exponential-phase cultures of Δ*CCNA_02386* (panel D) or Δ*CCNA_03538* (panel E) infected at MOI 0.01 in PYE. Unadsorbed phage remaining in the supernatant after 60 min was quantified and expressed as a percentage of the initial titer at time zero. Strains carried either an empty vector (EV) or a complementation plasmid expressing the deleted gene (++). Bars represent mean ± SD from two (**E**) or three (**C–D**) biological replicates. Statistical significance was assessed by multiple unpaired *t*-tests with Holm–Šídák correction for panel **C** (*P* < 0.05; ns, not significant) and by one-way ANOVA with Tukey’s multiple-comparisons test for panels **D** and **E** (****P < 0.0001; ns, not significant).

To systematically identify additional host genes required for Circe infection, we performed a phage challenge experiment using a randomly barcoded transposon (RB-TnSeq) library of *C. crescentus* CB15. The library was infected with CirceC at an MOI of 1, and barcode abundance was quantified at multiple time points across the infection cycle (Fig. 7A). As expected, transposon insertions in the *CCNA_03538* epimerase were highly enriched by 7.5 h post-infection, consistent with the strong resistance phenotype observed in both spontaneous mutant and in-frame deletion strains (Fig. 7B; Table S3). In addition, we observed late enrichment of transposon mutants with insertions in multiple genes required for LPS O-polysaccharide biosynthesis, including *CCNA_00497*, *CCNA_01068*, *CCNA_02386*, *CCNA_03733*, and *CCNA_03744* (*rfbB*); these genes have been previously implicated in assembly or export of smooth lipopolysaccharide (S-LPS) (36, 37) (Fig. 7; Table S3). Several of these mutants also exhibited mild fitness defects at early time points, potentially reflecting increased vulnerability to phage infection stress (in cases where mutants become infected). This result may also simply reflect the slower growth of these mutants at earlier time points before the culture density begins to drop. By late time points (7.5 h), however, the large competitive advantage of mutants with disrupted S-LPS or other envelope polysaccharides is evident. To further confirm that S-LPS genes are important host factors for Circe infection, we tested susceptibility to CirceC in strains bearing in-frame deletions in *CCNA_00497*, *CCNA_02386*, and *rfbB*. Each mutant was resistant to infection in liquid-based assays (Fig. 7C). Genetic complementation with each corresponding gene from a xylose-inducible promoter restored phage sensitivity. To examine if S-LPS is important for the binding of Circe to the cell surface, we performed an adsorption assay using the Δ*CCNA_02386* mutant*.* After 60 min of incubation, we observed no adsorption of phage to the Δ*CCNA_02386* strain. Circe adsorption to this strain could be restored via genetic complementation (Fig. 7D). Together, these results confirm that smooth LPS is a key host contributor to the adsorption of CirceC. Importantly, even though strains lacking *CCNA_03538* are resistant to CirceC (Fig. 6), the phage adsorbs efficiently to the ∆*CCNA_03538* mutant (Fig. 7E), suggesting that the contribution of CCNA_03538 activity to productive phage infection occurs after phage attachment.

We further identified a distinct group of genes that showed fitness advantages at early time points post-infection that decreased by 7.5 h, suggestive of host functions that impact the early stages of infection or that are more relevant at low phage titer (Fig. S2). This class included genes from the sphingolipid biosynthesis and export cluster spanning *CCNA_01210* to *CCNA_01226* (38–40). Specific mutants in this cluster, including *CCNA_01210* (phosphoglycerate cytidylyltransferase), *CCNA_01212* (ceramide synthase), and *CCNA_01220* (serine palmitoyltransferase), showed an interesting fitness profile with modest but reproducible fitness gains that peaked at 4.5 h but returned to near zero by 7.5 h. These results indicate that sphingolipid biosynthesis impacts CirceC infection through a yet-to-be-determined mechanism. It is known that *C. crescentus* lipid A is dispensable under select genetic conditions if sphingolipids are present (37). The relationship between the *C. crescentus* sphingolipid biosynthesis gene cluster, LPS O-polysaccharide synthesis, and susceptibility to phage Circe is an interesting future area of investigation.

Our genome-scale screen also revealed a group of genes that exhibited basally reduced fitness scores across all time points, including the 0 h time point (Table S3), likely reflecting a general growth defect. These genes included CCNA_01427 (*bamE*), a lipoprotein subunit of the β-barrel assembly machinery (BAM complex) previously shown to have basally low fitness scores in Tn-seq studies (36, 41, 42); cell envelope regulator CCNA_01817 (*ntrX*) (43); CCNA_00050 (*lnt*), which catalyzes the final step in outer membrane lipoprotein maturation, and CCNA_02037 (*lon*), encoding an ATP-dependent protease involved in general proteostasis.

## DISCUSSION

### On the significance of sequence variation in Circe gene gp063

Circe is a member of the *Schitoviridae* family and, to our knowledge, represents the first N4-like phage reported to infect *Caulobacter*. We isolated two Circe variants (CirceC and CirceH) that differ by a single non-synonymous mutation in the gene gp063. A PSI-BLAST search identified homologs of gp063 in several *Caulobacter* and *Bradyrhizobium* genomes, as well as in the temperate *Caulobacter* phage S2B, but did not identify gp063 orthologs in other N4-like phage genomes. In some genomes, gp063 homologs have been generically annotated as tail fiber proteins. However, we have found no experimental evidence that gp063 or its homologs contribute to tail fiber structure or function in any phage. To further explore the possible function of gp063, we generated a predicted structure using AlphaFold3 (44) (Fig. S3A) and queried it against the BFVD repository (45) using FoldSeek (46). This analysis identified structural similarity to several Caudovirales phage proteins, which share low sequence identity (~20%) with gp063 but exhibit highly similar predicted tertiary structures. gp063 did not share significant structural homology with any proteins of known function in other structural databases accessed by Foldseek.

Although CirceC and CirceH adsorbed to *C. crescentus* with similar efficiency, CirceH displayed delayed and biphasic clearing kinetics, suggesting that the gp063 mutation affects a post-adsorption stage of the infection cycle (e.g., genome ejection, replication, or lysis regulation). Our structural predictions indicate that the C-terminal domain of gp063 contains five α-helices, with five phenylalanine residues buried within a hydrophobic core (Fig. S3A and B). In CirceH, one of these stacked phenylalanines (F91) is replaced by an isoleucine (Fig. S3B). Because many phage structural proteins (including tail-associated factors) function as oligomers, we additionally used AlphaFold3 to model gp063 as a trimer and found that this oligomeric state yielded the most coherent assembly among those tested (Fig. S3C). However, the predicted interface was only moderately supported (ipTM ~0.55), and we did not observe notable differences between the CirceC and CirceH trimer models. Consistent with this, the conservative F→I substitution is not predicted to destabilize the overall fold, though we cannot rule out subtle effects on oligomerization or inter-subunit contacts that could influence gp063 function and contribute to the observed plaquing and infection differences.

Although it remains unclear which Circe variant represents the ancestral or predominant form in nature, the presence of two gp063 alleles that yield phages with distinct infection phenotypes suggests potential for adaptive diversification at this locus. The coexistence of CirceC and CirceH aligns with established phage strategies for modulating host range, such as phase variation at tail fiber loci that alters expression or structural properties (47, 48) or the use of diversity-generating retroelements to modify receptor-binding proteins (49). It is plausible that *Caulobacter* phage Circe populations experience selection on gp063 variants that enable adaptation to changes in local receptor landscapes or that help maintain fitness in the face of other host population shifts.

### Circe employs a host engagement strategy characteristic of other N4-like phages

To identify host factors required for productive infection by *Caulobacter* phage CirceC, we combined forward genetic selection with genome-wide RB-TnSeq screening. These approaches identified multiple host genes that strongly shape susceptibility to Circe, with many of the strongest hits clustering in carbohydrate metabolism, particularly genes involved in S-LPS biosynthesis and export. Disruption of select S-LPS pathway genes conferred complete resistance to Circe and caused a marked defect in adsorption, supporting a model in which one or more S-LPS components function as the primary adsorption receptor. This conclusion is consistent with known host-engagement mechanisms of other N4-like phages. For example, *E. coli* phage N4 initially binds an exopolysaccharide produced by NfrB before transitioning to the outer membrane protein NfrA as a terminal receptor (50–53). Similarly, lipopolysaccharide has been implicated in host interaction for N4-like phages infecting *Achromobacter* and *Shigella* species (54, 55). Together, these parallels suggest that recognition of envelope polysaccharides may be a conserved feature of N4-like phage infection across diverse hosts, and our results extend this model to *C. crescentus*.

In *C. crescentus*, S-LPS also anchors the paracrystalline surface layer (S-layer) protein RsaA (56), which forms a highly ordered envelope surface structure (57). Although S-layers have been proposed to shield phage receptors in other systems, our RB-TnSeq screen did not reveal a significant fitness impact of *rsaA* mutation during Circe challenge (Table S3), though genes that are directly adjacent to *rsaA* on the chromosome and that are associated with S-layer biogenesis do influence Circe infection (Fig. S2). These results indicate that the S-layer *per se* is not required for Circe infection and instead supports a model in which Circe engages S-LPS directly. This distinguishes Circe from φCr30, a *Caulobacter* phage that is reported to use RsaA as its receptor (18).

In addition to S-LPS biosynthesis and export genes, we identified the WcaG-like NAD-dependent epimerase/dehydratase CCNA_03538 as a strong determinant of Circe susceptibility. An in-frame *CCNA_03538* deletion mutant did not exhibit a phage adsorption defect but remained fully resistant to Circe. This separation of phenotypes suggests that CCNA_03538 is not required for initial attachment but instead contributes to a post-adsorption step that is essential for productive infection. One possibility is that CCNA_03538-dependent sugar metabolism modifies an envelope feature that Circe must recognize after initial S-LPS-mediated engagement, analogous to multi-step receptor usage observed for other N4-like phages (50–53). Alternatively, CCNA_03538 may influence the abundance or structure of a host surface determinant in a way that is not captured by our bulk adsorption assay but is nevertheless critical for infection progression.

Overall, our study defines a previously unrecognized phage–host interaction in which an N4-like phage exploits envelope polysaccharides to infect *C. crescentus*. This expands the known repertoire of *Caulobacter* phages and highlights Circe as a potential molecular tool for probing cell surface organization and envelope biogenesis in this widely used model system.

## MATERIALS AND METHODS

### Strains and growth conditions

*E. coli* was grown in Lysogeny broth (LB) or LB agar (1.5% wt/vol) at 37°C (58). Medium was supplemented with the following antibiotics when necessary: kanamycin 50 µg mL^−1^ or chloramphenicol 20 µg mL^−1^. *C. crescentus* was grown in peptone-yeast extract (PYE) broth (0.2% [wt/vol] peptone, 0.1% [wt/vol] yeast extract, 1 mM MgSO_4_, 0.5 mM CaCl_2_) or PYE agar (1.5% wt/vol) at 30°C (59). Solid medium was supplemented with kanamycin 25 µg mL^−1^ when necessary. Natural freshwater from which *Caulobacter* phage Circe was isolated was collected off the western public dock of Lake Lansing in Haslett, Michigan, USA (42.755,534 latitude, −84.404864 longitude).

### Plasmid and strain construction

Plasmids were cloned using standard molecular biology techniques and the primers listed in Table S4. For overexpression constructs, inserts were cloned behind the cumate-inducible promoter (P_Q5_) in pPTM057, which integrates at the xylose locus (*xylX*) (60). For deletion constructs, inserts were constructed with overlap PCR with regions upstream and downstream of the target gene and cloned into pNPTS138. Clones were confirmed by Sanger sequencing. Plasmids were transformed into *C. crescentus* via triparental mating (59). Transformants were selected by plating on PYE agar supplemented with the appropriate antibiotic and nalidixic acid to select against *E. coli* donor strains. Gene deletions were constructed via a standard two-step recombination/counterselection method using *sacB* as the counterselection marker. Transformants from the triparental mating were incubated in PYE broth without selection at 30°C for 8 h. Cultures were plated on PYE supplemented with 3% (wt/vol) sucrose to select for recombinants that had lost the plasmid. Mutants were confirmed via PCR amplification of the target gene from sucrose-resistant, kanamycin-sensitive clones.

### Transmission electron microscopy

Phages were prepared and imaged at the Michigan State University Center for Advanced Microscopy. Briefly, 10 µL concentrated phage was incubated on a formvar carbon-coated grid for 5 min, washed with distilled water, and excess water was blotted away with Whatman filter paper. Grids were stained with 1% uranyl acetate and imaged on a 1400 Flash JEOL transmission electron microscope. Phage capsid diameters were measured from vertex-to-vertex in FIJI, and the three vertex-to-vertex measurements for each capsid were averaged.

### Efficiency of plating assays

Strains were inoculated in PYE and incubated at 30°C overnight. Overnight cultures were diluted 1/10 in PYE and incubated at 30°C for ~4 h, then diluted to 0.2 OD_660_. For plating, 5 mL PYE top agar (0.3% [wt/vol] agar), 1 mL *C. crescentus* strain (0.2 OD_660_), and 150 μL 30% (wt/vol) xylose were mixed and poured on PYE plates. For images, phage stocks were diluted to 1 × 10^9^ PFU/mL, serial dilutions were prepared from the stock, and 5 μL dilutions were plated on PYE top agar. Plates were incubated at 30°C overnight and imaged. For EOP quantification, phage stocks at 1 × 10^9^ PFU/mL were serially diluted, and 100 µL undiluted or 9.1 × 10^−7^ diluted sample was plated on PYE top agar. Plates were incubated at 30°C for 18 h, and plaques were enumerated. Statistical analysis was carried out in GraphPad 10.6.0.

### Clearing assay

Strains were inoculated in PYE supplemented with 50 μM cumate or 0.15% (wt/vol) xylose when applicable and incubated at 30°C overnight. Overnight cultures were diluted 1/10 in PYE supplemented with 50 μM cumate or 0.15% (wt/vol) xylose when applicable and incubated at 30°C for ~3–7 h. Cultures were diluted to 0.1 OD_660_ (~6.7 × 10^7^ cells/mL) and CirceC or CirceH were added to 0.1 MOI (6.7 × 10^6^ PFU/mL), 1 MOI (6.7 × 10^7^ PFU/mL), or 10 MOI (6.7 × 10^8^ PFU/mL). Cultures were incubated at 30°C for 24 h while shaking, and OD_660_ was measured. For clearing curves, 1 mL sample was added to three wells of a 24-well plate for technical replicates. Plates were shaken (Shaking mode: orbital; orbital frequency: 559 cpm [1 mm]; orbital speed: slow) at 30°C, and OD_660_ was measured every 15 min for 16 h in a Synergy HTX multi-mode plate reader (BioTek). For plate reader curves, the absorbance for three wells with PYE (i.e., blanks) was averaged and subtracted from wells that contained cells to get the blank-corrected value. Each biological replicate data point is the average absorbance of three blank-corrected technical replicates. Statistical analysis was carried out in GraphPad 10.6.0.

### Phage adsorption assays

Strains were inoculated in PYE supplemented with 50 μM cumate or 0.15% (wt/vol) xylose when applicable and incubated at 30°C overnight. Overnight cultures were diluted 1/10 in PYE and incubated at 30°C for ~4 h. Cultures were diluted to 0.2 OD_660_ (~1.3 × 10^8^ cells/mL), 1.3 × 10^6^ PFU/mL (0.01 MOI) Circe was added, and cultures were incubated at 30°C while shaking. At 0, 7.5, 15, 30, 45, and 60 min post-infection, samples were taken and diluted 1/100 in PYE, pelleted at 17,000 × *g* for 1 min, and 300 μL supernatant was mixed with 20 μL chloroform. For plating, 5 mL PYE top agar (0.3% [wt/vol] agar), 1 mL *C. crescentus* CB15 (0.2 OD_660_), and 150 μL 30% (wt/vol) xylose were mixed and poured on PYE plates. To determine the number of unadsorbed phage, samples were diluted where appropriate, and 100 μL was plated on PYE top agar. Plates were incubated at 30°C overnight, and plaques were enumerated. Percent unadsorbed phage was calculated as (PFU_t=x_ /PFU_t=0_) × 100. The adsorption constant was calculated using a nonlinear regression for the equation $P=P_{0}\times e^{-kNt}$, where *k* is the adsorption constant, *t* is time, *N* is the bacterial density, *P*_0_ is the starting phage concentration, and *P* is the final phage concentration. Statistical analysis was carried out in GraphPad 10.6.0.

### Genomic DNA isolation

For bacterial genomic DNA, strains were inoculated in PYE and incubated at 30°C overnight. Cultures were pelleted at 12,000 × *g* for 1 min, and supernatant was removed. Pellets were washed in 0.5 mL H_2_O, then resuspended in 100 μL TE (10 mM Tris, pH 8, 0.1 mM EDTA) with 20 ng/μL RNase A. A volume of 500 μL GES solution (5.08 M guanidium thiocyanate, 0.1 M EDTA, 0.5% [vol/vol] sarkosyl) was added, samples were vortexed, then samples were heated at 60°C for 15 min. A volume of 250 μL 7.5 M cold ammonium acetate was added, samples were vortexed for 15 s and incubated on ice for 10 min. A volume of 500 μL chloroform was added, samples were vortexed for 15 s, and pelleted at 12,000 × *g* for 10 min. The aqueous phase was transferred to a fresh tube, and 0.54 volumes of cold isopropanol were added. Samples were mixed by inversion and incubated at room temperature for 15 min. Samples were pelleted at 12,000 × *g* for 3 min, and supernatant was removed. Pellets were washed with 700 μL 70% (vol/vol) ethanol, then air-dried for 10 min. Pellets were resuspended in 100 μL TE buffer.

For phage genomic DNA, *C. crescentus* CB15 was inoculated in PYE and incubated at 30°C overnight. Cultures were diluted 1/10 in fresh PYE and inoculated at 30°C until the culture reached 0.1 OD_660_. Circe was added to (0.1 MOI), and cultures were incubated at 30°C for 24 h. Cultures were pelleted at 7,197 × *g* for 5 min, and the supernatant was transferred to a fresh tube. Circe was concentrated in an Amicon Ultra 15 (30K) concentrator, then treated with RNase A and Turbo DNase for 60 min at 37°C. EDTA was added to a final concentration of 15 mM, then the samples were incubated at 95°C for 15 min. Lysozyme (0.1 mg/mL final concentration) and SDS (0.5% final concentration) were added, and samples were incubated at 55°C for 60 min. An equal volume of chloroform was added, samples were vortexed, and samples were pelleted at 12,000 × *g* for 10 min. The aqueous phase was transferred to a fresh tube, and a second chloroform extraction was performed. Ammonium acetate (2.5 M final concentration) was added to the aqueous phase, samples were mixed by inversion, and 0.6 volumes of isopropanol was added. Samples were mixed by inversion, then stored at −20°C for 60 min. Samples were pelleted at 17,000 × *g* for 5 min at 4°C, and supernatant was removed. Pellets were washed with 700 μL 70% (vol/vol) ethanol, then air-dried for 10 min. Pellets were resuspended in 50 μL 10 mM Tris, pH 8.5 buffer.

### Phage genome assembly and analysis

Library preparation and sequencing of genomic DNA from Circe were performed by SeqCoast. Libraries were prepared with the Illumina DNA Prep tagmentation kit and IDT For Illumina Unique Dual Indexes and sequenced on the Illumina NextSeq2000 platform to get 150 bp paired-end reads. Reads were trimmed with BBDuk2 (length: >100 bp; QC: >30) and assembled using Unicycler (61) (version 0.5.1) on Galaxy (bridging mode: normal; lowest k-mer size: 0.2; highest k-mer size: 0.95; k-mer steps in assembly: 10; filter out contigs lower than this fraction of chromosomal depth: 0.25). The physical ends of the chromosome were determined through Sanger sequencing. The Circe genome was annotated with a combination of Pharokka (version 1.7.3) (62), Pyrodigal (63), Phold (version 0.2.0; https://github.com/gbouras13/phold), and Phynteny (version 0.1.13; https://github.com/susiegriggo/Phynteny) using Google Colab. The viral proteomic tree was constructed using the ViPTree webserver (version 4.0) (31). Putative spanins were identified using SpaninDB (64); signal peptides were identified using SignalP (65). Sequence reads used to assemble the *Caulobacter* phage Circe genome are available through NCBI BioProject accession PRJNA1345760. The phage Circe genome accession number is PX630703.1.

### Whole-genome resequencing and polymorphism identification

Library preparation and sequencing of genomic DNA from phage-resistant backgrounds were performed by SeqCoast. Libraries were prepared with the Illumina DNA Prep tagmentation kit and IDT For Illumina Unique Dual Indexes and sequenced on the Illumina NextSeq2000 platform to get 150 bp paired-end reads. Reads were mapped to the *C. crescentus* NA1000 reference genome (GenBank accession CP001340), and polymorphisms were identified with Breseq (66).

### RB-TnSeq to identify host genes required for Circe infection

We used a previously described and characterized RB-TnSeq mutant library in *C. crescentus* CB15 (36, 41) to identify genes important for infection by the N4-like phage Circe. For each experiment, a 1 mL aliquot of the mutant library was inoculated into 22 mL of PYE medium and grown shaking at 30°C overnight. For each experiment, a 7 mL overnight culture was diluted into 60 mL PYE and grown at 30°C with shaking until reaching the early-exponential phase (OD_660_ ~0.1). At this point, CirceC phage was added at an MOI of 1.0, and cultures were returned to 30°C with aeration.

Samples were collected at 0 (uninfected), 1.5, 3, 4.5, and 7.5 h post-infection. A 10 mL of culture for the 0, 1.5, 3, and 4.5 h samples or 20 mL of culture for the 7.5 h samples was removed, pelleted by centrifugation (12,000 × *g*, 3 min, 4°C), washed once with 1 mL PYE, and stored as a cell pellet at –20°C. Genomic DNA was extracted and used for barcode amplification following the method of Wetmore et al. (67). Briefly, cell pellets were resuspended in 10–20 μL sterile water and used as a template for PCR amplification of barcodes using Q5 polymerase (New England Biolabs) in 20 μL reaction volumes. Each reaction contained 1× Q5 buffer, 1× GC enhancer, 0.8 U Q5 polymerase, 0.2 mM dNTPs, 0.5 μM each of the universal forward primer (Barseq_P1) and a unique indexed reverse primer (Barseq_P2_ITxxx). Thermocycling conditions were 98°C for 4 min; 25 cycles of 98°C for 30 s, 55°C for 30 s, and 72°C for 30 s; followed by 72°C for 5 min and hold at 4°C.

PCR products were pooled and subjected to single-end 50-bp sequencing on an Illumina NovaSeq using TruSeq primers. Barcode sequences have been deposited in the NCBI Sequence Read Archive under BioProject accession PRJNA1345765. Barcode counts were processed and analyzed using the FEBA pipeline as described in Wetmore et al. (67). Briefly, barcode counts from each post-infection sample were compared to the reference (t = 0) barcode counts to compute fitness scores (log₂ ratio of barcode abundance) for each mutant. Statistical t-scores, which take into account variance in fitness of each transposon insertion strain for each gene (67), reflect the mean fitness of a gene divided by a standard error metric. This statistic accounts for variability in fitness measurements across mutant strains and provides some indication of how many standard-error units the fitness effect differs from zero.

### Host infection fitness time-course clustering

Gene fitness scores (*t*-scores) were obtained at 1.5, 3.0, 4.5, and 7.5 h following N4-like phage (CirceC) infection. Genes with |*t*| ≥4 at one or more time points were retained for analysis. To compress dynamic range while preserving magnitude and sign, a sign-preserving logarithmic transformation was applied. Hierarchical clustering of genes (rows) was performed on the transformed matrix using Euclidean distance and average linkage through SciPy. Gene order was determined by dendrogram leaf order. Time points (columns) were not clustered and are displayed chronologically. Complete RB-TnSeq fitness scores and corresponding t-scores are presented in Table S3.

## SUPPLEMENTAL MATERIAL

Supplemental figures jb.00488-25-s0001.pdf Figures S1 to S3.

Table S1 jb.00488-25-s0002.xlsx Circe annotations from Phold and Phynteny.

Table S2 jb.00488-25-s0003.xlsx BreSeq analysis of polymorphisms in spontaneous Circe-resistant mutant strains.

Table S3 jb.00488-25-s0004.xlsx RB-TnSeq (BarSeq) scores for genes in a *Caulobacter crescentus* CB15 randomly barcoded transposon mutant library across a Circe infection time course.

Table S4 jb.00488-25-s0005.xlsx Strains and plasmids.

ASM does not own the copyrights to Supplemental Material that may be linked to, or accessed through, an article. The authors have granted ASM a non-exclusive, world-wide license to publish the Supplemental Material files. Please contact the corresponding author directly for reuse.
