# Supplementary material for: An N4-like Caulobacter phage requires host smooth lipopolysaccharide biosynthesis for infection
Source: J Bacteriol. 2026 Feb 25;208(3):e00488-25. doi: 10.1128/jb.00488-25 (PMC13001222; doi:10.1128/jb.00488-25)
Supplement: Supplemental figures — Figures S1 to S3. [file jb.00488-25-s0001.pdf]

Supplemental Material

An N4-like *Caulobacter* phage requires host smooth lipopolysaccharide biosynthesis for infection

Maeve McLaughlin[1], Katheren Barger[1], Charlotte Barron[1], Makena Fisher[1], Larissa Kohn[1], Priscilla Mac-Kittah[1], Aretha Fiebig[2], Sean Crosson[2]

[1]Department of Biology, University of Michigan-Flint, Flint, MI, USA

[2]Department of Microbiology, Genetics, and Immunology, Michigan State University, East Lansing, MI, USA

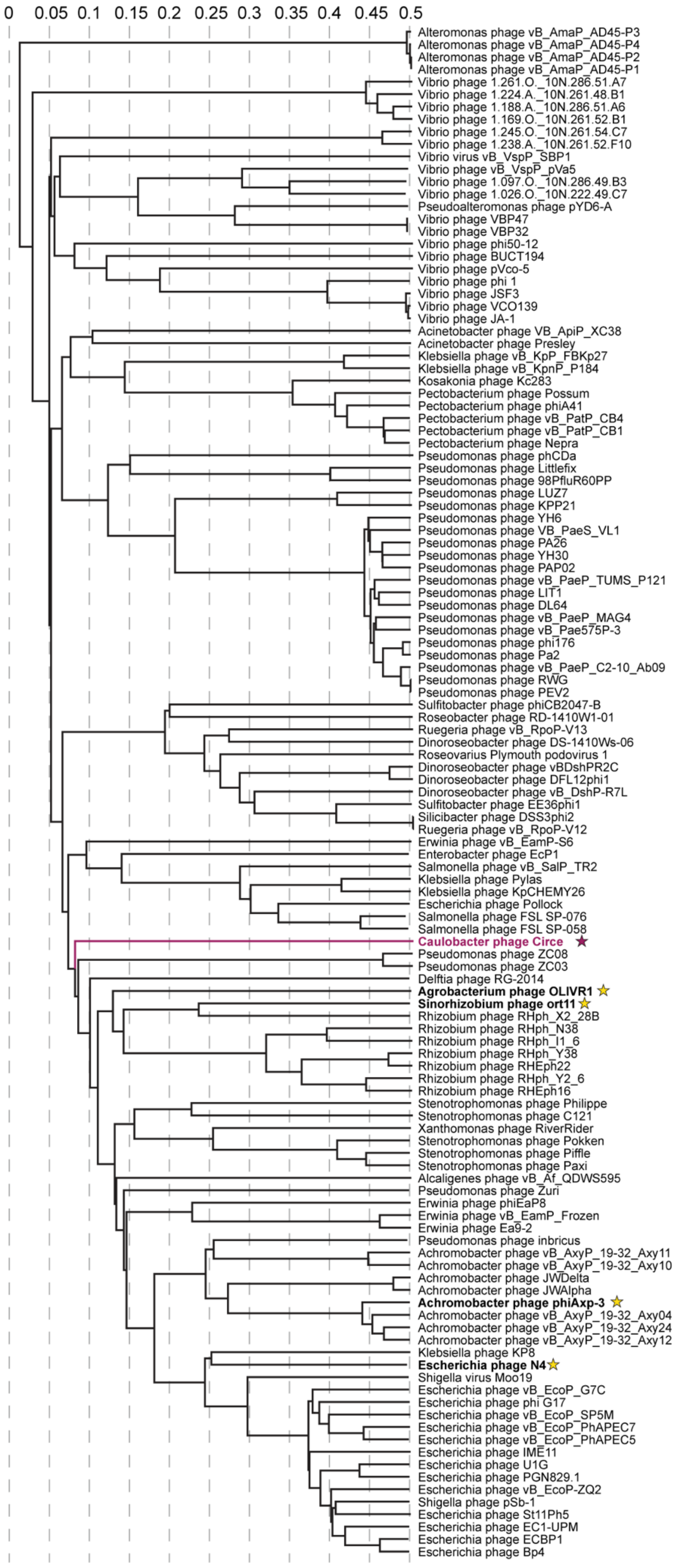
0 0.05 0.1 0.15 0.2 0.25 0.3 0.35 0.4 0.45 0.5
Alteromonas phage vB_AmaP_AD45-P3
Alteromonas phage vB_AmaP_AD45-P4
Alteromonas phage vB_AmaP_AD45-P2
Alteromonas phage vB_AmaP_AD45-P1
Vibrio phage 1.261.O._10N.286.51.A7
Vibrio phage 1.224.A._10N.261.48.B1
Vibrio phage 1.188.A._10N.286.51.A6
Vibrio phage 1.169.O._10N.261.52.B1
Vibrio phage 1.245.O._10N.261.54.C7
Vibrio phage 1.238.A._10N.261.52.F10
Vibrio virus vB_VspP_SBP1
Vibrio phage vB_VspP_pVa5
Vibrio phage 1.097.O._10N.286.49.B3
Vibrio phage 1.026.O._10N.222.49.C7
Pseudoalteromonas phage pYD6-A
Vibrio phage VBP47
Vibrio phage VBP32
Vibrio phage phi50-12
Vibrio phage BUCT194
Vibrio phage pVco-5
Vibrio phage phi 1
Vibrio phage JSF3
Vibrio phage VCO139
Vibrio phage JA-1
Acinetobacter phage VB_ApiP_XC38
Acinetobacter phage Presley
Klebsiella phage vB_KpP_FBKp27
Klebsiella phage vB_KpnP_P184
Kosakonia phage Kc283
Pectobacterium phage Possum
Pectobacterium phage phiA41
Pectobacterium phage vB_PatP_CB4
Pectobacterium phage vB_PatP_CB1
Pectobacterium phage Nepra
Pseudomonas phage phCDa
Pseudomonas phage Littlefix
Pseudomonas phage 98PfluR60PP
Pseudomonas phage LUZ7
Pseudomonas phage KPP21
Pseudomonas phage YH6
Pseudomonas phage VB_PaeS_VL1
Pseudomonas phage PA26
Pseudomonas phage YH30
Pseudomonas phage PAP02
Pseudomonas phage vB_PaeP_TUMS_P121
Pseudomonas phage LIT1
Pseudomonas phage DL64
Pseudomonas phage vB_PaeP_MAG4
Pseudomonas phage vB_Pae575P-3
Pseudomonas phage phi176
Pseudomonas phage Pa2
Pseudomonas phage vB_PaeP_C2-10_Ab09
Pseudomonas phage RWG
Pseudomonas phage PEV2
Sulfitobacter phage phiCB2047-B
Roseobacter phage RD-1410W1-01
Ruegeria phage vB_RpoP-V13
Dinoroseobacter phage DS-1410Ws-06
Roseovarius Plymouth podovirus 1
Dinoroseobacter phage vBDshPR2C
Dinoroseobacter phage DFL12phi1
Dinoroseobacter phage vB_DshP-R7L
Sulfitobacter phage EE36phi1
Silicibacter phage DSS3phi2
Ruegeria phage vB_RpoP-V12
Erwinia phage vB_EamP-S6
Enterobacter phage EcP1
Salmonella phage vB_SalP_TR2
Klebsiella phage Pylas
Klebsiella phage KpCHEMY26
Escherichia phage Pollock
Salmonella phage FSL SP-076
Salmonella phage FSL SP-058
Caulobacter phage Circe
Pseudomonas phage ZC08
Pseudomonas phage ZC03
Delftia phage RG-2014
Agrobacterium phage OLIVR1
Sinorhizobium phage ort11
Rhizobium phage RHph_X2_28B
Rhizobium phage RHph_N38
Rhizobium phage RHph_I1_6
Rhizobium phage RHph_Y38
Rhizobium phage RHEph22
Rhizobium phage RHph_Y2_6
Rhizobium phage RHEph16
Stenotrophomonas phage Philippe
Stenotrophomonas phage C121
Xanthomonas phage RiverRider
Stenotrophomonas phage Pokken
Stenotrophomonas phage Piffle
Stenotrophomonas phage Paxi
Alcaligenes phage vB_Af_QDWS595
Pseudomonas phage Zuri
Erwinia phage phiEaP8
Erwinia phage vB_EamP_Frozen
Erwinia phage Ea9-2
Pseudomonas phage inbricus
Achromobacter phage vB_AxyP_19-32_Axy11
Achromobacter phage vB_AxyP_19-32_Axy10
Achromobacter phage JWDelta
Achromobacter phage JWAlpha
Achromobacter phage phiAxp-3
Achromobacter phage vB_AxyP_19-32_Axy04
Achromobacter phage vB_AxyP_19-32_Axy24
Achromobacter phage vB_AxyP_19-32_Axy12
Klebsiella phage KP8
Escherichia phage N4
Shigella virus Moo19
Escherichia phage vB_EcoP_G7C
Escherichia phage phi G17
Escherichia phage vB_EcoP_SP5M
Escherichia phage vB_EcoP_PhAPEC7
Escherichia phage vB_EcoP_PhAPEC5
Escherichia phage IME11
Escherichia phage U1G
Escherichia phage PGN829.1
Escherichia phage vB_EcoP-ZQ2
Shigella phage pSb-1
Escherichia phage St11Ph5
Escherichia phage EC1-UPM
Escherichia phage ECBP1
Escherichia phage Bp4

Figure S1. Viral proteomic dendrogram generated using ViPTree (http://www.genome.jp/viptree) showing the relationship between Circe and 122 related N4-like genomes. Circe is marked with a red line and red star. N4-like phage compared in Figure 2B are bolded and marked with a yellow star.

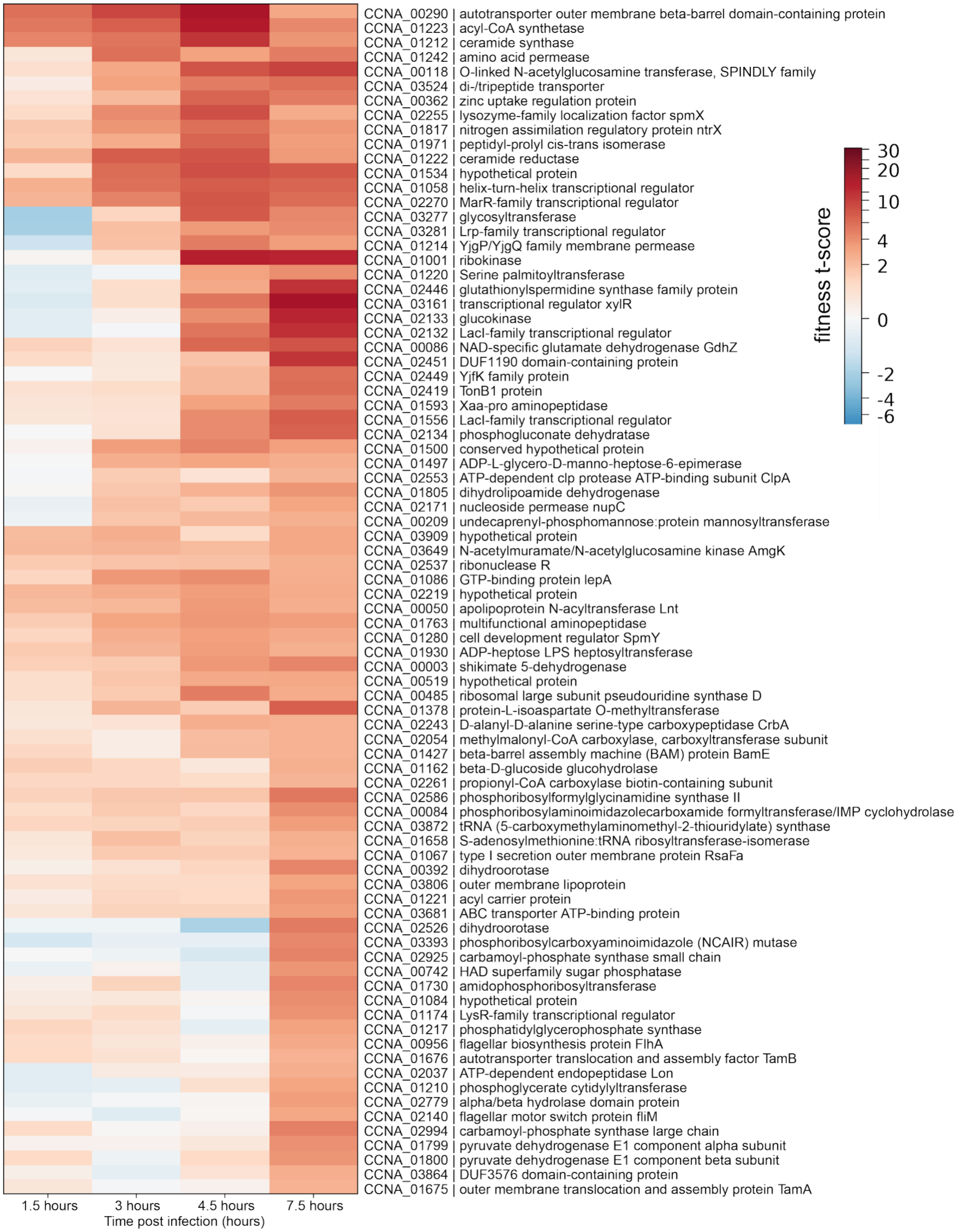

Figure S2. Hierarchically clustered heatmap of *C. crescentus* gene fitness t-scores at 1.5, 3.0, 4.5, and 7.5 h post-infection with CirceC at MOI=1. Rows include genes with | t |≥ 4 at ≥1 time point. Figure shows clustered genes not presented in panel 7B. Complete fitness data are presented in Table S3.

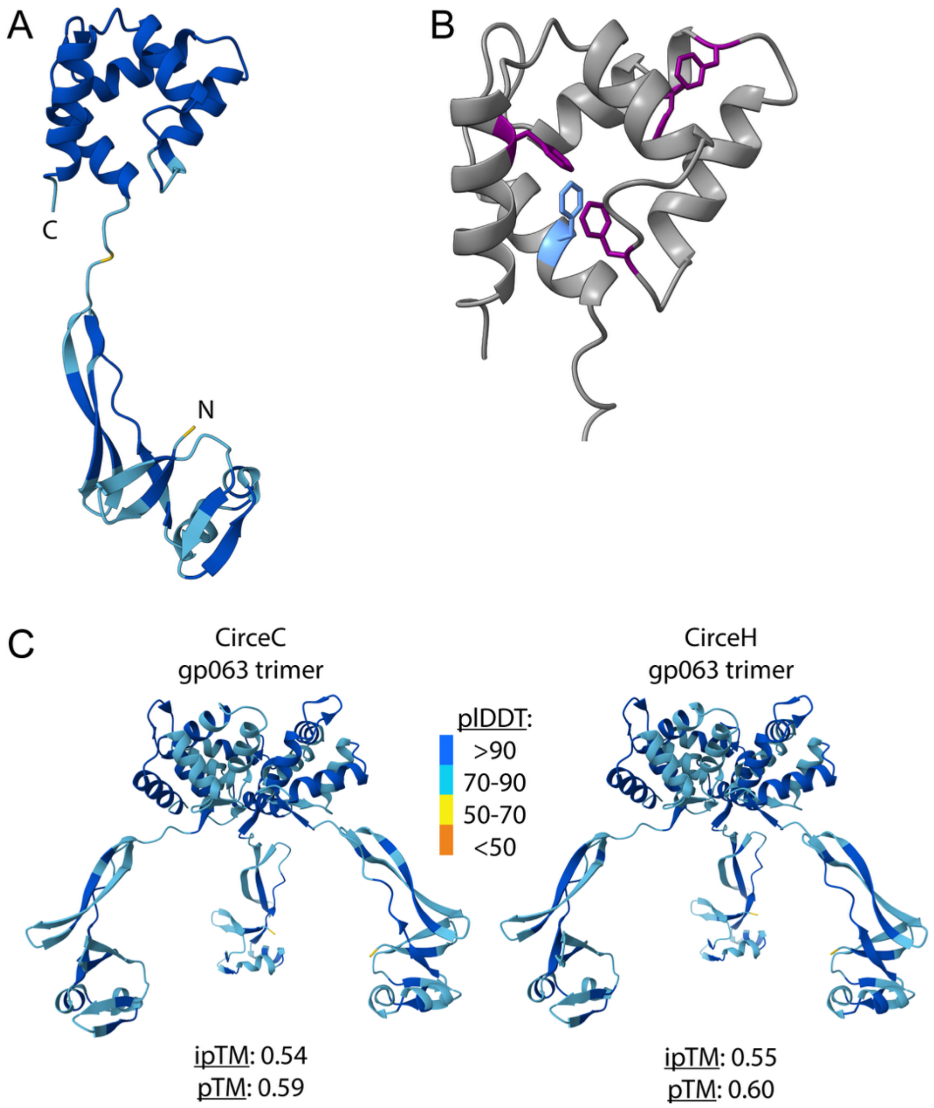

Figure S3. Predicted gp063 structure. Structure of gp063 predicted by AlphaFold3. A) Full-length gp063 colored based on pIDDT score (pTM=0.53). B) C-terminal domain of gp063. Phenylalanine residues are colored purple. Phenylalanine 91 is colored blue. C) Prediction of gp063 trimer folding in CirceC (left) and CirceH (right).

Supplemental Tables

Table S1. Circe automated gene annotations

Table S2. BreSeq analysis of polymorphisms in spontaneous Circe-resistant mutant strains.

Table S3. RB-TnSeq (BarSeq) scores for genes in a *C. crescentus* CB15 randomly barcoded transposon mutant library upon Circe infection

Table S4. Strain table
